# Impact of surface disinfection with hydrogen peroxide on the prevalence of vancomycin-resistant enterococci (VRE) in hospital wards

**DOI:** 10.3205/dgkh000348

**Published:** 2020-06-22

**Authors:** Anna Häring, Ursel Heudorf, Martin Exner, Frank-Albert Pitten, Oliver Waidmann, Daniel Hack, Volkhard A. J. Kempf, Claudia Reinheimer

**Affiliations:** 1 Institute of Medical Microbiology and Infection Control, University Hospital Frankfurt, Frankfurt am Main, Germany; 2 Public Health Department of the City of Frankfurt am Main, Germany; 3 Institute for Hygiene and Public Health, University Hospital Bonn, Germany; 4 Institute for Hospital Hygiene and Infection Control, Giessen, Germany; 5 Department of Internal Medicine I, University Hospital Frankfurt, Frankfurt am Main, Germany; 6 University Center of Infectious Diseases, University Hospital Frankfurt, Frankfurt am Main, Germany; 7 University Center of Competence for Infection Control Frankfurt – Giessen – Marburg, Frankfurt am Main, Germany

**Keywords:** vancomycin-resistant enterococci, infection control, hydrogen peroxide, hospital environment, Hawthorne effect

## Abstract

**Objective:** Vancomycin-resistant *enterococci* (VRE) are of major concern in infection control. Although broad infection control actions to check VRE have been implemented, VRE remain part of daily infection prevention in clinical settings. Cleaning procedures in the inanimate ward environment might play a key role in controlling VRE. In order to optimize infection control management at University Hospital Frankfurt, Germany (UHF), this study evaluates the impact of H_2_O_2_-containing cleaning wipes compared to Glucoprotamin containing wipes on VRE prevalence in intensive care wards.

**Methods:** Retrospective analyses were conducted of the VRE prevalence on environmental materials obtained from three intensive care units (ICU) at UHF for 17 months prior to (T1) and during the 25 months after (T2) the implementation of H_2_O_2_-containing cleaning wipes from January 2016 to June 2019. The bactericidal power of the two disinfectants against VRE was compared using the 4-field test according to EN 16615 (2015).

**Results:** At T1 and T2, n=666 and n=710 environmental samples, respectively, were obtained. At T1, 24.2% (n=161/666; 95% confidence interval: 21.0–27.6) and at T2, 6.9% (n=49/710; 5.1–9.0) samples were positive for VRE. *In vitro* disinfectant testing did not reveal any superiority of H_2_O_2_ over glucoprotamin. No effect on the VRE prevalence in patients’ rectal screening materials was observed.

**Conclusion:** Though Glucoprotamin and H_2_O_2_ were *in vitro* equally effective against VRE, the prevalence of VRE in ICU environment at UHF decreased after implementation of H_2_O_2_-containig wipes. This might be due to multiple factors, of which we consider the impact of the *Hawthorne effect* to be the strongest. Success of infection control strategies might depend on the compliance of the persons critically involved. Transparent information on infection control strategies is suggested to increase compliance and should therefore be considered both in daily infection control and outbreak management.

## Zusammenfassung

**Hintergrund:** Vancomycin-resistente Enterokokken (VRE) stellen zunehmend eine krankenhaushygienische Herausforderung dar. Wenngleich für VRE bereits umfangreiche Empfehlungen zur Infektionsprävention implementiert wurden, bleibt VRE weiterhin ein fester Bestandteil der täglichen Infektionsprävention im klinischen Setting. Reinigungs- und Desinfektionsmaßnahmen der unbelebten Stationsumgebung könnten dabei eine Schlüsselrolle bei der Eindämmung von VRE darstellen. Mit dem Ziel, eine optimierte krankenhaushygienische Herangehensweise zur Eindämmung von VRE am Universitätsklinikum Frankfurt (UKF) zu schaffen, untersucht diese Arbeit den Einfluss von H_2_O_2_-enthaltenden im Vergleich zu Glucoprotamin-enthaltenden Reinigungstüchern auf die VRE-Prävalenz in Umweltmaterialien am UKF.

**Methodik:** Retrospektive Analyse der VRE-Prävalenz in Umweltmaterialien dreier Intensivstationen (ITS) am UKF 17 Monate vor (T1) und in den ersten 25 Monate nach (T2) Umstellung auf H_2_O_2_-enthaltende Wischtücher zur Oberflächendesinfektion (Zeitraum: Januar 2016 bis Juni 2019). Mittels 4-Felder-Tests (EN 16615; 2015) Vergleich von H_2_O_2_ und Glucoprotamin bezüglich ihrer Wirksamkeit auf VRE.

**Ergebnis:** In T1 und T2 wurden n=666 bzw. n=710 Umweltmaterialien untersucht. In T1 wurde VRE in 24,2% der Materialien (n=161/666; 95%-Konfidenzintervall: 21,0–27,6) und in T2 in 6,9% (n=49/710; 5, –9,0) nachgewiesen. Die Desinfektionsmitteltestung *in vitro* erbrachte keine Überlegenheit von H_2_O_2_ gegenüber Glucoprotamin. Die VRE-Prävalenz in Screeningmaterialien von am UKF behandelten Patienten derselben Zeiträume blieb unverändert.

**Schlussfolgerung:** Die VRE-Prävalenz in Umweltmaterialien der ITS am UKF nahm seit Umstellung auf H_2_O_2_-getränkte Wischtücher zur Oberflächendesinfektion signifikant ab. Hinsichtlich der antimikrobiellen Wirksamkeit zeigten sich im 4-Felder-Test äquivalente Ergebnisse für Glucoprotamin und H_2_O_2_. Die dargestellte Dynamik scheint daher auf multifaktorielle Aspekte zurückzuführen zu sein, wovon wir den Einfluss des *Hawthorne Effect* am stärksten einschätzen. Der Erfolg von Strategien zur Infektionsprävention hängt nach unserer Erfahrung essenziell von der Compliance der involvierten Personen ab. Diese kann durch eine transparente Informationspolitk im täglichen krankenhausyhgienischen Geschäft wie auch im Ausbruchsfalle gestärkt werden.

## Introduction

Vancomycin-resistant *enterococci* (VRE) are globally of major concern in terms of infection control and public health. Although broad infection control actions have been implemented, daily infection prevention measures must still deal with VRE. VRE are an important cause of morbidity and mortality and have been shown to increase the economic burden for hospitals when compared to vancomycin-susceptible isolates [1], [2], [3], [4], [5], [6]. In Europe, the proportion of invasive *Enterococcus* *faecium* isolates with resistance to vancomycin is inhomogeneous, with Germany ranking in the upper middle range [7]. Even within Germany, the proportion of VRE bloodstream infections varies greatly, with the highest percentages reported from the four federal states of North-Rhine Westphalia, Hesse, Thuringia and Saxony, which form the “VRE belt” [8], [9]. The epidemiology of VRE, however, is complex. This might be reflected by the increasing proportion of VRE in nosocomial infections due to *enterococci* as well as a gradual widening of the belt in Germany [8], [9], [10]. Although the reasons for this epidemiological phenomenon remain unclear, the National Public Health agency of France reports that glycopeptide resistant *enterococci* were most frequently reported from Ile-de-France, Lorraine and Nord-Pas-de-Calais [11], which might suggest that the belt stretches both over France and Germany. For the Rhine-Main area in Hesse, with almost three million inhabitants, the circulation of a single VRE clone (MLST type ST117, cgMLST complex type CT71 with a common vanB chromosomal insertion site) has been shown [12].

Located in the center of Hesse, University Hospital Frankfurt (UHF) has thus been facing one of the highest background VRE prevalences in Germany for several years. This is illustrated by the dramatically high number of cases (1,000 on average) annually of newly detected VRE in samples obtained from any patient body site. As the background prevalence remains unchangeable, the infection control strategy at UHF focuses intensely on the prevention of nosocomial VRE infection. UHF therefore offers a range of periodic and intensive tutorials, for instance addressing the epidemiology and current scientific status of multidrug-resistant organisms (MDRO), e.g., methicillin-resistant *Staphylococcus aureus* (MRSA), multidrug-resistant gram-negative organisms (MDRGN), and VRE. These tutorials also address basic infection control strategies, e.g., hand hygiene, cleaning and disinfection procedures for patients’ and wards’ inanimate environments, which have been shown to play a key role in the spread of MDRO [13], [14], [15], [16], [17]. A small outbreak at UHF (five patients affected) of *Klebsiella pneumoniae* with carbapenem resistance in spring 2017 [18] impelled a change from glucoprotamin to H_2_O_2_-containing wipes for surface disinfection. This resulted in a continuous decline of VRE prevalence in material obtained from ICU environmental sites at UHF. Our findings therefore might encourage discussion of ways of fighting VRE in hospital settings.

## Material and methods

### Study setting and observation period

This study retrospectively investigated the VRE prevalence in near-patient inanimate environments in three wards at UHF before (T1) and after (T2) changing the wards’ environmental cleaning procedure in June 2017. The observation period T1 included the 17 months from January 2016 to May 2017, and T2 included the subsequent 25 months until June 2019. 

Environmental specimens were taken by the same sampler over the entire observation period of 42 months.

### Ward environmental cleaning procedure in T1 and T2

In June 2017, environmental cleaning procedures underwent a major change on three wards at UHF: one surgical ICU (ward 1), one internal medicine ICU (ward 2) and one surgical intermediate care unit (ward 3). This change was a result of outbreak management of *K. pneumoniae* with carbapenem resistance at UHF in spring 2017 [18]. Ward environmental cleaning procedures during T1 were performed using the antimicrobial disinfectant glucoprotamin (Incidin^TM^ Plus Wipes, Ecolab, Monheim am Rhein, Germany) for surface disinfection. Since June 2017 (T2), ward environmental cleaning has been performed using a H_2_O_2_-based antimicrobial disinfectant (Incidin^TM^ OxyWipe S, Ecolab, Monheim am Rhein, Germany). 

During the observation period, no other new infection control interventions were implemented. The infection control protocol at UHF requires surfaces in patient rooms to be disinfected at least once daily and, in case of visible contamination, immediately. Furthermore, devices used daily, e.g. keyboards, computer mouse, ECG unit, need to be thoroughly wiped down with a disinfectant wipe after each use. Final disinfection of patients’ rooms upon discharge or transfer of patients should include all surfaces, inventory, and equipment. Cleaning procedures are carried out by nursing and cleaning staff at UHF. 

Any positive VRE result is immediately reported to the ward’s senior physician as well as to the ward’s medical and nursing on-duty hygiene officer. They are informed of the location where VRE has been detected and are asked to carry out prompt disinfection of the respective device; they are also required to report this result at the ward’s next team meeting and confirm proper execution of these actions as soon as it they have been performed. 

### Wards without permanent implementation of H_2_O_2_-based surface disinfection

In order to compare these findings with environmental samples obtained from wards on which the change from glucoprotamin to H_2_O_2_-containing wipes for surface disinfection was accomplished after than June 2017, we additionally evaluated the VRE prevalence in two wards which only temporarily changed to H_2_O_2_-based surface disinfection (ward N). These phases of temporary change to H_2_O_2_-containing wipes were

1. three weeks in November/December 2017 and
2. four weeks in March/April 2019.

Both episodes were due to non-availability of glucoprotamin at UHF. To analyze VRE prevalence after these episodes, the first sample round was taken and evaluated four weeks after the switch back to glucoprotamin.

In case of positive VRE results, the same cascade described above was initiated.

### Sampling and detection of VRE

In Germany, Infection Protection Law (Infektionsschutzgesetz; IfSG) determines several aspects of infection control, one of these being the mandatory epidemiological surveillance of organisms with multidrug resistance, such as VRE. As required by §23 IfSG [19], measures need to be taken in order to prevent the transmission of healthcare-associated pathogens, which also includes VRE. At UHF, this legal requirement is fulfilled *inter alia* by smear samples taken from inanimate environment, e.g., keyboards, drug-containing trolleys, medical devices or bandaging material, ECG units or ultrasound devices. The routine environmental smears are taken monthly from defined sites on the wards. All microbiological procedures were performed under quality-assured conditions (accredited standards according to ISO 17025:2005; certificate number D-PL-13102-01-00, valid through 2021) using cotton swabs (Süsse, Gudensberg, Germany) to take environmental smear samples, and were rubbed into tryptic soy broth plus lecithine/polysorbat 80 (Tween^®^ 80)/histidine/Nathiosulphate disinhibitor (LTHTh; Merck, Darmstadt, Germany). In case of turbidity within 48 hours of incubation at 36°C, the suspension was inoculated onto the CHROMID^®^ VRE plate (bioMérieux, Nürtingen, Germany) and incubated for a further 48 hours at 36°C. *E. faecium* were identified by matrix-assisted laser desorption ionisation – time of flight analysis (MALDI-TOF; VITEK MS; bioMérieux). Antibiotic susceptibility testing was performed according to international standard guidelines (Clinical and Laboratory Standards Institute) with VITEK 2 and antibiotic gradient tests (bioMérieux), as previously described [5].

### VRE prevalence in patients’ rectal screening samples

In addition, we retrospectively analyzed the prevalence of community-acquired (CA) VRE cases as well as VRE detected within the first three days after admittance, with the day of admittance being day one [20]; following the definition by the hospital-infection-surveillance system (Krankenhaus-Infektions-Surveillance-System, KISS) in accordance with the regulations of the National Reference Center for Surveillance of Nosocomial Infections, Berlin, Germany (Nationales Referenzzentrum für Surveillance von nosokomialen Infektionen). VRE cases detected beyond the first three days after admittance are categorized as “nosocomial detected (ND)”. Since “nosocomial” has often mistakenly been supposed to be equivalent to “hospital-acquired”, we recommend to use the terminus “nosocomial detected”. The limitations of the current criteria to characterize “nosocomial” will be discussed below. 

Patients admitted to any ICU at UHF are routinely screened for VRE and other pathogens. Screening for VRE includes a rectal swab on the day of admission and repetitive VRE screening on a fixed-day weekly routine. This fixed-day weekly screening consists of all patients being treated on the ward on this specific day. This procedure is backed up by recommendations of the Commission for Hospital Hygiene and Infection Prevention (KRINKO) at the Robert Koch Institute on VRE [21], the weekly routine screening at UHF therefore even exceeds this. 

This evaluation is based on the results from rectal screening materials obtained from gastroenterological patients admitted to ward 2 in the 12 weeks prior to the outbreak of *K. pneumoniae* with carbapenem resistance at UHF in spring 2017 (T1; January 2017 to March 2017; [18]) as well as the 12 weeks following the completion of outbreak management (T2; June 2017 to August 2017). These patient groups and the periods were chosen for several reasons.

1. Ward 2 has the highest patient VRE prevalences at UHF, which predetermines it to observe any VRE prevalence changes, however minor.
2. The gastroenterological patients admitted to ward 2 are similar in terms of medical treatment: they are all likely to present after a long history of medical pre-treatment, which implies similar exposure to antibiotic (selective) pressure in their medical history.
3. We therefore assume that the VRE prevalence in patient screening materials has largely been stable.

In order to estimate the patients’ VRE prevalence in ward 2, an observation period of 12 weeks seems to be appropriate to approach the patients’ base VRE prevalence. In order to assess the patients’ VRE prevalence in the H_2_O_2_ cleaning area, the observation period must be started as soon as possible after completion of the outbreak management. In order to exclude any over- or underestimation of VRE prevalence since implementation of H_2_O_2_, we chose an observation period of 12 weeks, which is equivalent to the period prior to the H_2_O_2_ implementation. In order to calculate the ratio of community acquired cases:nosocomial detected (CA:ND) for VRE cases, only data from patients having had ≥2 rectal screenings for VRE were included. This procedure guarantees exclusion of patients who had only one screening, and whose status regarding nosocomial detection of VRE cannot be assessed by only one single VRE screening on the day of admission. For periods T1 as well as T2, the patients’ number of weekly routine rectal screenings for VRE are assessed, which is an direct indicator for the duration of stay on ward 2. For the periods T1 and T2, the patients’ VRE cases are calculated as the CA:ND ratio. 

### Disinfectant testing

The disinfectants used during the two study periods were either 0.5% glucoprotamin (Incidin^TM^ Plus Wipes, Ecolab, Monheim am Rhein, Germany) or 1.5% H_2_O_2_-based pre-soaked wipes (Incidin^TM^ OxyWipe S, Ecolab, Monheim am Rhein, Germany). Both products are registered in the VAH List of Disinfectants with valid certificates indicating sufficient antimicrobial efficacy against the test organisms used, according to the European standards for the testing of disinfectants [22]. Given that *E. faecium* is one of the compulsory gram-positive test organisms, relevant differences regarding the efficacy of the two products are unlikely. Therefore, we decided to compare the bactericidal activity of both products in the 4-field test according to EN 16615:2015 [22] using three different strains *E. faecium* [23]. 

All tests were carried out at high organic burden (soiled conditions) using 0.3% albumin and 0.3% sheep erythrocytes as contaminants. To this end, the Institute for Hospital Hygiene and Infection Control (iki) in Giessen, Germany obtained the following strains from UHF: (a) *E. faecium* ATCC 19434, (b) *E. faecium* VRE-RV69, and (c) *E. faecium* (vancomycin-resistant) as environmental isolates from UHF. Strain (c) was randomly obtained from routine laboratory testing at the Department for Infection Control at UHF.

### Statistical analysis

For statistical analysis, the biostatistical data file from the University of Münster, Germany, was used [24]. 95% confidence intervals (95%CI) were calculated based on binomial distribution and p-values (2-tailed) of p≤0.05 were considered statistically significant. 

## Results

### Wards 1–3: period prior to implementation of H_2_O_2_-containing cleaning wipes (January 2016–May 2017; T1)

**Figure 1:**
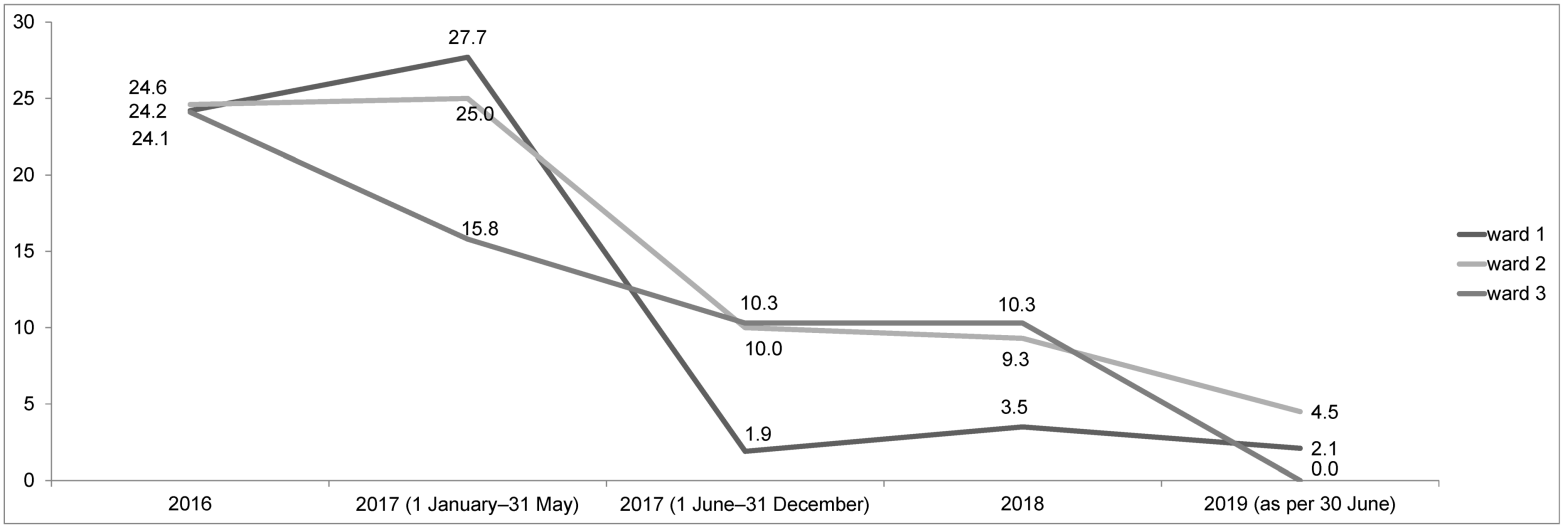
Changing VRE prevalence in the respective ward’s environmental samples over time (T1: Jan 2016–May 2017; T2: June 2017–June 2019); respective ward’s data are given in boxes

Within T1, a total of n=666 samples were obtained from ICU environments at UHF. Overall, n=161 of these (24.2%; 21.0–27.6) tested positive for VRE. Regarding the individual wards, the prevalence of VRE in the environment of wards 1, 2 and 3 was 25.1% (n=42/167; 18.8–32.4), 24.7% (n=80/324; 20.1–29.8) and 22.3% (n=39/175; 16.4–29.2), respectively. Further details are shown in Figure 1 (Fig. 1).

### Wards 1–3: period since implementation of H_2_O_2_-containing cleaning wipes (June 2017–June 2019; T2)

Within T2, a total of n=710 samples were obtained from ICU environments at UHF. Overall, n=49 of these (6.9%; 5.1–9.0) tested positive for VRE, which is significantly lower than the overall value of T1 (p<0.05). Regarding the individual wards, the prevalence of VRE in the environment of ward 1 was 1.9% (n=1/54), 3.5% (n=3/86) and 2.1% (n=1/48) in 2017 (June to December), in 2018 and in 2019 (January to 30 June), respectively. On ward 2, the VRE prevalence continuously dropped as well, with 10.0% (n=12/120), 9.3% (n=15/162) and 4.5% (n=3/66) in the same periods. A decline was also seen for ward 3, with 10.4% (n=5/48), 10.3% (n=9/87) and 0.0% (n=0/39) in the respective periods. Further details are shown in Figure 1 (Fig. 1).

### Wards 1–3: Total VRE prevalence in ward environmental samples in the transition phase of 2017

**Figure 2:**
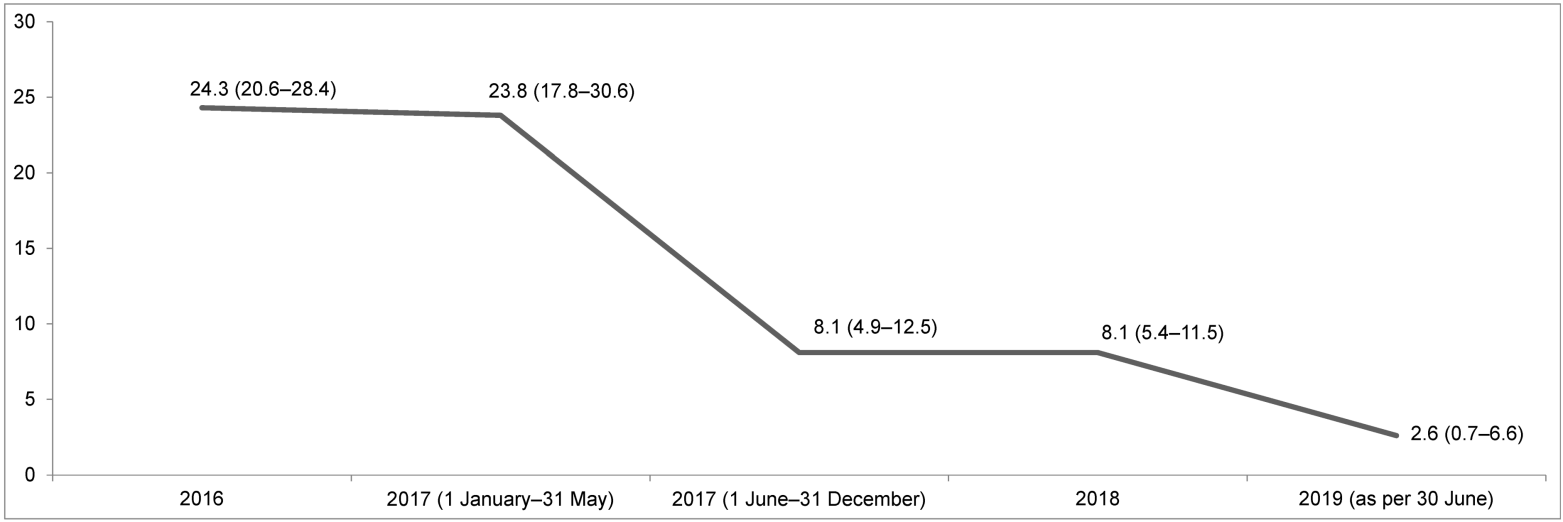
Overall ward VRE prevalence in environmental samples over time (T1: Jan 2016–May 2017; T2: June 2017–June 2019); exact data are given in boxes as *p*-value (95% confidence intervals)

As shown in Figure 2 (Fig. 2), the total VRE prevalence in the ward environmental samples peaked in T1 with 24.3% (20.6–28.4) in entire year 2016 and 23.8% (17.8–30.6) in 2017 (January to May), with a sharp, significant decline to 8.1% (4.9–12.5; p=0.000034) after the implementation of H_2_O_2_-containing cleaning wipes in June 2017 (including June to December 2017). 

### VRE prevalence in wards without permanent change to H_2_O_2_-containing cleaning wipes

**Figure 3:**
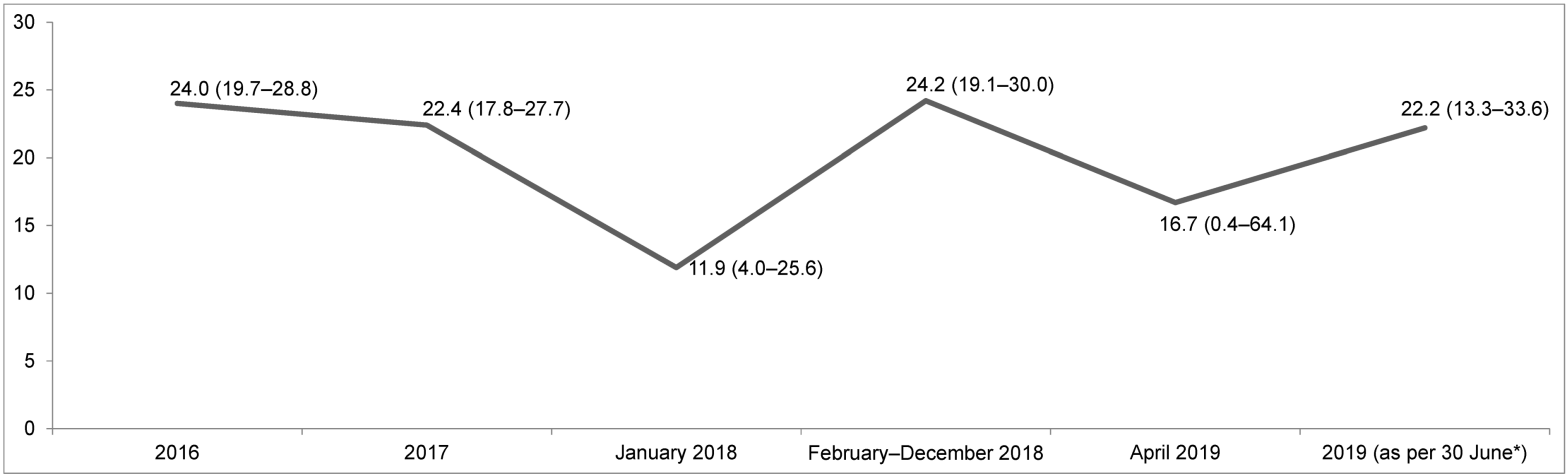
VRE prevalence in environmental samples obtained from two wards, which temporarily changed from glucoprotamin to H_2_O_2_-containing cleaning wipes for surface disinfection in November/December 2017 and March/April 2019; exact data are given in boxes with 95% confidence intervals; (*) April 2019 excluded

As illustrated in Figure 3 (Fig. 3), VRE prevalence in theses wards’ environmental samples amounted to an average of 23.2% in 2016 and 2017. After the first change in November/December 2017, the VRE prevalence dropped to 11.9% (4.0–25.6) in January 2018. Afterwards, it rose again to 24.2% (19.1–30.0) overall between February and December 2018. After the second change in March/April 2019, it declined again to 16.7% (0.4–64.1). Concerning the remaining samples in 2019 (as per 30 June 2019) with April being excluded (due to the change in March/April 2019, see above), the VRE prevalence was 22.2% (13.3–33.6). None of the changes in VRE prevalence on these wards were significant (p>0.05).

### VRE cases in patients’ rectal screening samples, ratio of CA:ND

With regard to T1, the median number of weekly screenings was n=3. The ratio CA-VRE:ND-VRE was 33.3:66.7. In T2, the median number of weekly screenings was n=2. The ratio of CA-VRE:ND-VRE was 36.4:63.6. No significant differences were observed.

### Results of disinfectant testing

The results obtained at 2 or 5 min time of action are presented in Table 1 (Tab. 1). The strains (a), (b) and (c) were as mentioned above.

The data indicate that there was no difference regarding the test product (glucoprotamin vs. H_2_O_2_), the test organism (strain 1, 2 or 3) or the time of action (2 vs. 5 min). All the controls were valid as demanded by EN 16615. However, even if differences may exist at lower concentrations of the test products, none were observed when the products were tested under conditions quite similar to their application on UHF wards.

## Discussion

The German VRE belt is formed by North-Rhine Westfalia, Hesse, Thuringia and Saxony [8], [9], [10]. Thus, University Hospital Frankfurt is centrally located in this area of high background VRE prevalence and, as a result of this exposure, faces almost n=1,000 of newly detected VRE cases per year in screening and clinical samples obtained from patients admitted to UHF. As part of daily routine infection control, VRE is therefore continually addressed in hygiene and cleaning training courses as well as seminars for employees at UHF. Despite the continuous efforts of all staff, the VRE prevalence in environment material was ca. 24% at UHF between January 2016 and May 2017 (T1).

In June 2017, however, the environmental disinfectant procedure changed from glucoprotamin to H_2_O_2_-containing cleaning wipes in three intensive care units (Figure 1 (Fig. 1)), which resulted from the management of an outbreak at UHF [18]. Afterwards, the VRE prevalence in environmental material sharply declined to 8.1% in 2018 and 2.6% in 2019 (as of 30 June 2019; Figure 2 (Fig. 2)). This was also observed for wards which had only temporarily changed from glucoprotamin to H_2_O_2_-containing cleaning wipes (Figure 3 (Fig. 3)).

This dynamic is remarkable, since H_2_O_2_ has not proven to be superior to glucoprotamin in terms of efficacy against VRE. Therefore, one or possibly a combination of factors is suggested to be responsible for this trend. In the phase immediately following the change and in the aftermath of outbreak management at UHF in spring 2017 [18], we propose that the observed decline of VRE prevalence was due to the* Hawthorne effect*, which might have led to behavioral changes among staff involved in infection control interventions and might thus have encouraged this trend [25], [26], [27]. As shown in other infection control studies, however, this effect is only temporary [27], [28], [29], indicating that the VRE prevalence in environmental samples at UHF might be due to additional factors.

One of these might be the practical handling of glucoprotamin compared to H_2_O_2_-containing cleaning wipes. Glucoprotamin-containing wipes are not supplied in a ready-to-use form. Before being operational, the dry wipes need to be unpacked and placed in a dispenser. Then, 2.5 liters of Incidin^TM^ disinfection solution must be poured into the dispenser and the first wipe needs pulled through the dispenser’s lid. After closing the dispenser, the disinfection solution must be allowed to be absorbed by the wipes. Only when wipes are completely saturated are they ready to use, which takes up to 60 minutes (manufacturer’s information) [30]. In contrast, the H_2_O_2_-containing wipes are supplied pre-assembled, saturated and ready for immediate use, which is more user-friendly [29], [31]. 

Furthermore, subjective sensations might additionally contribute to this trend. Whereas the scent of glucoprotamin-containing wipes is almost neutral, the scent of H_2_O_2_ wipes is distinctive and might be characterized by some as acrid. This subjective sensation might have increased the users’ compliance [32] – similar to the phenomenon that the perceived effect of drugs depends on their color [33], [34]. These two effects are supposed to buttress the Hawthorne effect and might therefore have additionally contributed to the decline of the VRE prevalence in the environmental samples throughout the entire period from June 2017 to June 2019 (T2). 

Whereas prior environmental contamination has clearly been shown to increase the risk of acquiring VRE [35], the VRE prevalence in environmental samples, however, might hypothetically also depend on the total VRE incidence at UHF. However, the number of newly detected VRE was around n=1,000 annually at UHF in 2016, 2017, and 2018. In 2019, the number of newly detected VRE was about n=380 as of 31 May 2019, resulting in an estimated number (based on a linear course) of around n=910 for the entire year 2019. Given that this amount approximately equals the number of newly detected VRE of the previous years, VRE incidence at UHF has been stable and therefore cannot have affected the number of VRE in samples obtained from ward environments. Additionally, since samples were always taken by the same person (see Materials and methods), systematic bias is thus highly unlikely. 

Moreover, biofilms have been shown to be an excellent habitat for MDRO in hospital environments [36]. To manage such colonization, H_2_O_2_ vapor systems have been shown to reduce health care-associated infections in patients when used for terminal disinfection [37], [38]. Hence, H_2_O_2_ may have made biofilms more susceptible, thinner, or even more brittle than did glucoprotamin. In future research, this effect should be analyzed experimentally by quantifying the composition of such biofilms. 

Furthermore, the decrease in VRE incidence might have been caused by an enhanced awareness for infection control and hospital hygiene in general, e.g., through training seminars. For every occupational group at UHF, employees of the Department of Infection Control at UHF hold training seminars on infection control several times a year. Whereas the frequency of these courses was increased during the outbreak management during April and June 2017 [18], the type and quantity of the courses returned to a stable, pre-outbreak level for the rest of the observation period during July 2017 to June 2019. Therefore, multiple infection prevention initiatives were ongoing during the study period as part of a regular, routine bundle of infection control measures at UHF. The change of surface cleaning procedure was the only measure that changed during this time. Thus, the transient increase of training seminars during the outbreak in 2017 [18] might have initially contributed to the drop (Figure 1 (Fig. 1) and Figure 2 (Fig. 2)), but this specific outbreak-related training is highly unlikely to have affected the VRE prevalence in 2018 and 2019. 

The findings of this study might be limited by several factors. First, it was a single-center study and the results therefore can only be transferred to other hospitals to a limited extent (e.g., due to different local VRE prevalence, local screening procedures and medical profile of the institution). In particular, the profile of university medicine is clearly not comparable to the medical profile of a hospital providing only basic health care; university hospitals admit a high number of special-needs patients who are more likely to carry a MDRO, compared to the clientele of hospitals with a lower medical profile. 

Because at UHF we take swift infection-control action any time VRE are detected on environmental surfaces, independent of the number of colony forming units found, in this study, we did not determine the quantity of VRE in the environmental samples. Admittedly, this might have been necessary to more reliably quantify VRE contamination. In future studies, this aspect should be evaluated.

Regarding patients’ CA-VRE:ND-VRE ratio on ward 2, no effect was detected after the implementation of H_2_O_2_-containing cleaning wipes. However, this was not surprising, since the background prevalence of VRE is consistently high in the Rhine-Main region [8], [9], [10] and will not be affected by our change in ward cleaning management. To estimate any effect on the number of nosocomial cases, the data of all patients – those who had only one screening in particular – would be needed. In addition, data on the sensitivity of one single rectal screening for VRE are lacking up to now. Hence, it remains unclear whether a patient who screened negative upon admission to the hospital but in a subsequent positive screening after submission must be classified as “nosocomially detected” or as a slipped case in initial screening. These aspects should be addressed in further research, in which the medical profile of university hospitals should also be considered, as this is where patients with complex conditions, often protracted clinical histories after pre-treatment in various other hospitals, and long histories of antibiotic treatment are frequently admitted.

This, however, makes obvious the limitations of the current criteria for defining “nosocomial” as recommended by KISS and Centers for Disease Control and Prevention (CDC), as previously discussed [39]. Currently, “nosocomial” is defined in terms of time of the pathogen’s first detection with regard to the day of admittance. In case of the occurrence of a “resistance plasmid transfer” [40], the label “nosocomial” can also be highly problematic, as the transfer of a resistant plasmid from one species to another, e.g., *VanA* from VRE to *Staphylococcus aureus*, resulting in vancomycin-resistant *S. aureus* [40], might be misinterpreted as a new nosocomial acquisition. The prevalence of VRE in environmental materials has significantly decreased since switching from glucoprotamin to H_2_O_2_-containing cleaning wipes at UHF. Although H_2_O_2_ and glucoprotamin had equivalent efficacy against VRE, several other factors are supposed to be responsible. 

In conclusion, the initial effect might have been caused by a raised general awareness for infection control as a result of an outbreak managed at UHF in spring 2017 [18]. Since any positive VRE result is quickly reported to key players on the ward and prompt actions are required, the *Hawthorne effect* is suggested to have stabilized the adherence to infection control throughout the entire observation period. Thus, attenuation of the *Hawthorne effect* in the UHF setting seems to be unlikely. Handling or subjective perception, e.g., scent, might have also contributed to this effect. Additionally, increased self-efficacy among the staff might also have contributed to this trend. Based on our experience, adherence might be strengthened by transparently providing information on the sense and purpose of infection control strategies. We therefore suggest that this should be considered in both daily infection control routines and outbreak management.

## Notes

### Competing interests

The authors declare that they have no competing interests.

### Legal conditions and ethical approval

This study was conducted within the legal requirements given by German Infection Protection Law (Infektionsschutzgesetz) § 23 inter alia, and was additionally approved by the Ethics Board of the University Hospital Frankfurt, Germany (ethics votum No. E151/17).

## Figures and Tables

**Table 1:**
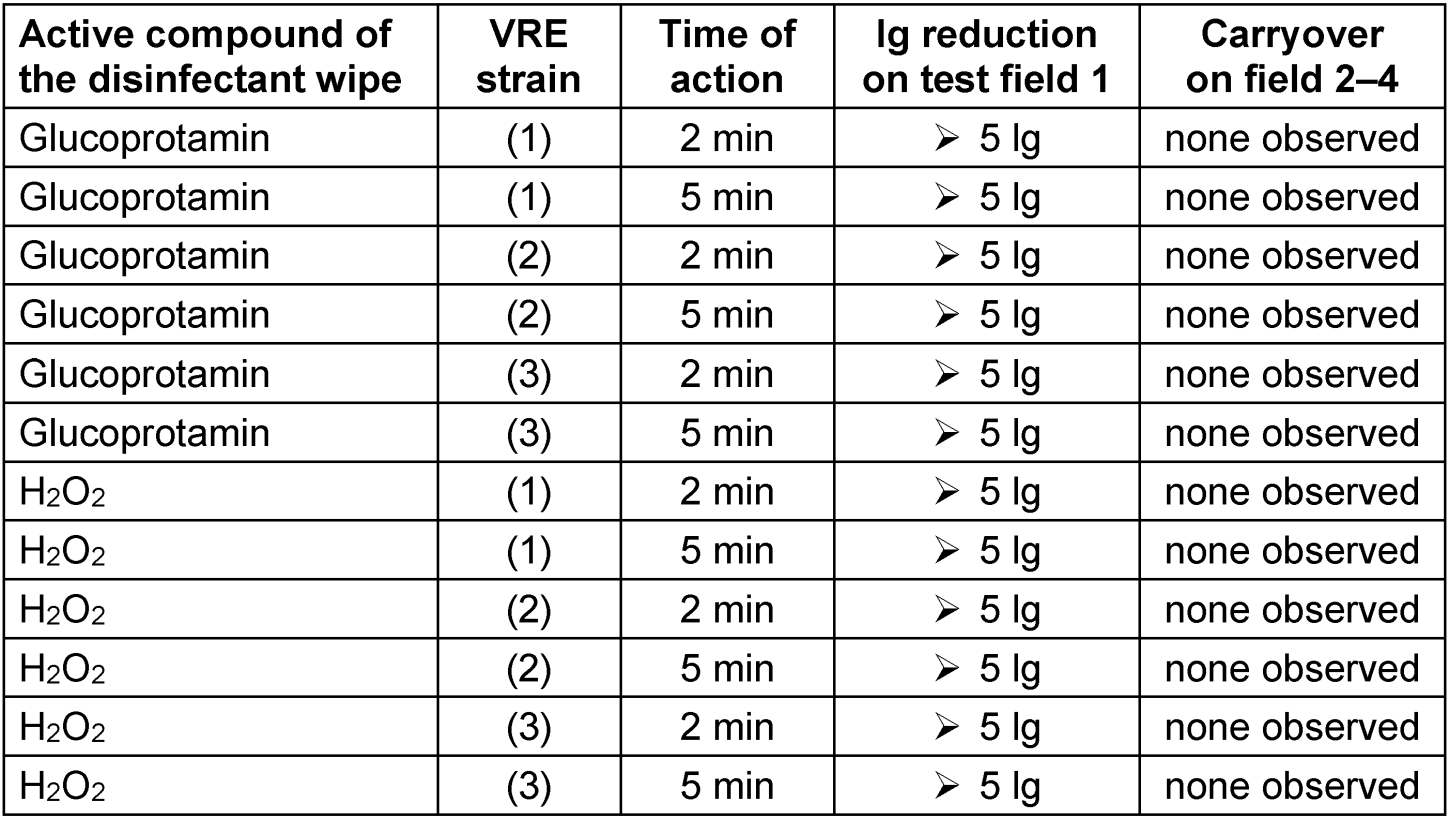
Disinfectant test results of glucoprotamin and H_2_O_2_-containing wipes for (1) *Enterococcus faecium* ATCC 19434, (2) *Enterococcus faecium* VRE-RV69 and (3) *Enterococcus faecium* environmental isolates
